# Construction of a plasmid-free l-leucine overproducing *Escherichia coli* strain through reprogramming of the metabolic flux

**DOI:** 10.1186/s13068-023-02397-x

**Published:** 2023-09-29

**Authors:** Yanan Hao, Xuewei Pan, Guomin Li, Jiajia You, Hengwei Zhang, Sihan Yan, Meijuan Xu, Zhiming Rao

**Affiliations:** 1 Key Laboratory of Industrial Biotechnology of the Ministry of Education, Laboratory of Applied Microorganisms and Metabolic Engineering, School of Biotechnology, Jiangnan University, Wuxi, 214122 China; 2 Yixing Institute of Food and Biotechnology Co., Ltd, Yixing, 214200 China

**Keywords:** l-Leucine, Metabolic engineering, Redox cofactors, Dynamic regulation, *Escherichia coli*

## Abstract

**Background:**

l-Leucine is a high-value amino acid with promising applications in the medicine and feed industries. However, the complex metabolic network and intracellular redox imbalance in fermentative microbes limit their efficient biosynthesis of l-leucine.

**Results:**

In this study, we applied rational metabolic engineering and a dynamic regulation strategy to construct a plasmid-free, non-auxotrophic *Escherichia coli* strain that overproduces l-leucine. First, the l-leucine biosynthesis pathway was strengthened through multi-step rational metabolic engineering. Then, a cooperative cofactor utilization strategy was designed to ensure redox balance for l-leucine production. Finally, to further improve the l-leucine yield, a toggle switch for dynamically controlling *sucAB* expression was applied to accurately regulate the tricarboxylic acid cycle and the carbon flux toward l-leucine biosynthesis. Strain LEU27 produced up to 55 g/L of l-leucine, with a yield of 0.23 g/g glucose.

**Conclusions:**

The combination of strategies can be applied to the development of microbial platforms that produce l-leucine and its derivatives.

**Supplementary Information:**

The online version contains supplementary material available at 10.1186/s13068-023-02397-x.

## Background

l-Leucine is a valuable functional amino acid, that is involved in many processes of cellular physiology and metabolism, including the signal molecules of protein metabolism, the maintenance of glucose homeostasis, and the regulation of lipid metabolism [1, 2]. The metabolic functions of l-leucine in regulating muscle protein synthesis and insulin release have high commercial value in the feed industry [3, 4]. Therefore, the increasing market demand for this amino acid has stimulated interest in the development of cost-effective approach for its production on an industrial scale [5]. Currently, mutagenesis and metabolic engineering strategies are the common methods used to engineer cellular factories for l-leucine production [6]. However, existing l-leucine fermentation systems are still too inefficient to achieve large-scale titers and economic competitiveness. Therefore, the construction of a superior microbial cell factory for l-leucine production is urgently required to meet future market demands for a sustainable supply.

By applying the strategies of systems metabolic engineering, researchers have made some progress in constructing l-leucine producing strains with *Corynebacterium glutamicum* as the “microbial chassis” [7]. Stemming from the precursor pyruvate, the l-leucine biosynthesis pathway involves seven reactions that are regulated by different mechanisms, including transcriptional attenuation and substrate inhibition [8]. In general, the superior l-leucine-producing performance of *C. glutamicum* is achieved through the promotion of glucose uptake, deletion of competitive consumption, and enhancement of the l-leucine biosynthesis pathway [9, 10]. Vogt et al. achieved a l-leucine titer (23.7 g/L in approximately 72 h) by increasing both the supply of the precursor and the feedback-resistance of 2-isopropylmalate [11]. However, despite the accumulation of l-leucine achieved with these above-mentioned strategies, the low titer and/or yield still limit translation of the process to the industrial scale. In addition, NADPH is also required in the l-leucine biosynthesis pathway and cofactor must be balanced to ensure efficient synthesis of l-leucine [12]. Wang et al. achieved an l-leucine production of 23.31 g/L by converting the cofactor requirements of l-leucine biosynthesis and glutamate dehydrogenase from NADPH to NADH [13]. However, by-products usually accumulated along with l-leucine, indicating that although redox balance is essential for maintaining strong flux in the l-leucine biosynthesis pathway, the by-products would inevitably result in a loss of productivity. Because the l-leucine biosynthesis pathway is long and sophisticated, and interlaces with intracellular redox imbalance, there is an urgent need for more efficient approaches to constructing redox balanced strains.

In addition to optimizing the carbon flux of the l-leucine biosynthesis pathway, enriching the precursor pool is another pivotal constraint to overproduction of the amino acid [14]. Because pyruvate and acetyl-CoA are the precursors of l-leucine, the synthesis of which is restricted by its coupling to cell growth, an undesired trade-off between biomass and products might be the crucial issues affecting the amino acid yield [15]. The promotion of l-leucine biosynthesis could be achieved by blocking or weakening the metabolic flux in the tricarboxylic acid (TCA) cycle. A prior effort to inhibit the TCA cycle had largely focused on reducing the activity of citrate synthase [11]. However, the metabolic valve regulated the TCA cycle statically, resulting in the premature loss of cell biomass, which is not the best strategy for l-leucine production. Recently, a quorum sensing (QS) circuit independent of inducers was applied for the efficient production of a variety of chemicals and dynamically changed the distribution of the carbon flux in the metabolic process [16]. In another study, inositol production was effectively increased through the design and use of a pathway-independent genetic control module to dynamically regulate the metabolic flux of glycolysis and redistribute the cellular metabolic network [17]. In addition, Jiang et al. (2019) successfully reduced the production cost of l-citrulline by combining modular engineering strategies with dynamic regulatory circuit to decouple the “growth mode” and “production mode” [18]. However, there are not many published studies on the use of dynamic regulation to increase the pyruvate supply and thereby l-leucine yield.

*Escherichia coli*, which carries the advantages of being susceptible to cultivate and having a short fermentation process, is another potential host for the industrial production of chemicals [19, 20]. In the present study, the combination of systems metabolic engineering and a dynamic regulation strategy was successfully implemented in *E. coli* to construct a strain capable of producing a high titer of l-leucine. First, the metabolic flux toward l-leucine biosynthesis was improved through the introduction of 2-isopropylmalate synthase variant and the overexpression of biosynthetic genes. The l-leucine transport system (including importer and exporter) was reprogrammed to achieve efficient l-leucine efflux. Next, redox balance in the metabolic network was achieved through the regeneration of NADPH and by changing the cofactor preference of l-leucine dehydrogenase, which resulted in the high-level synthesis of the target product. Finally, genetic control of the alpha-ketoglutarate dehydrogenase (*sucAB*) operon by QS was designed to dynamically regulate the glycolytic flux and reconstruct the metabolic flux of l-leucine. As a result of this modular combination strategy, the engineered *E. coli* strain (LEU27) produced 55 g of l-leucine in a 5 L bioreactor, with a maximum yield 0.23 g/g glucose. This method achieved the higher known level of l-leucine biosynthesis in *E. coli* and would be invaluable for the industrial-scale production of this high-value amino acid.

## Results and discussion

### Multi-step rational metabolic engineering for optimizing the l-leucine biosynthesis pathway

**Fig. 1:**
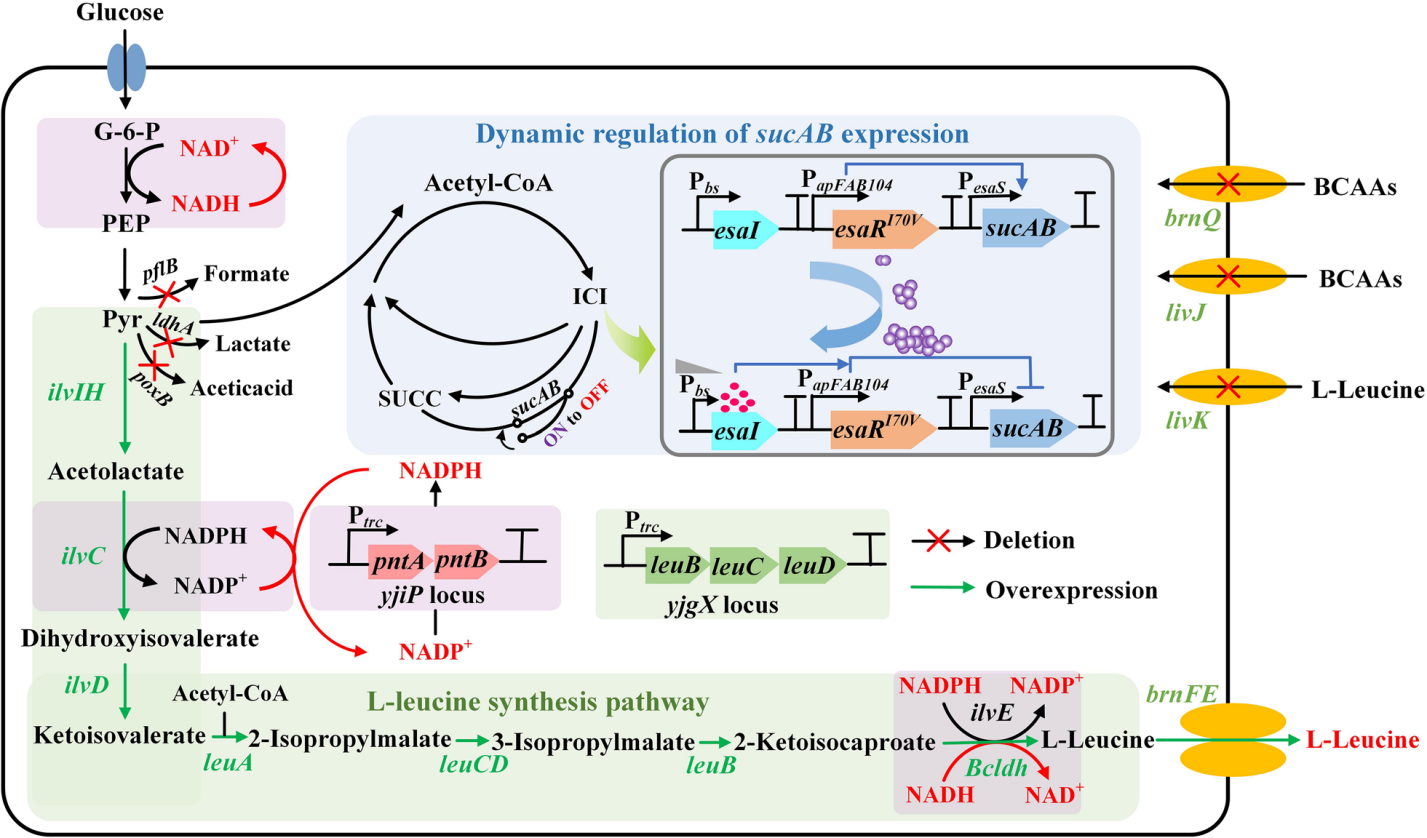
l-leucine biosynthesis pathway of *Escherichia coli*, and metabolic strategies for increasing the yield. G-6-P, glucose-6-phosphate; PEP, phosphoenol-pyruvic acid; Pyr, Pyruvate; SUCC, Succinate; ICI, Citrate; *ilvIH*, acetolactate synthase; *ilvC*, ketol-acid reductoisomerase; *ilvD*, dihydroxy-acid dehydratase; *ilvE*, branched-chain amino-acid aminotransferase; *leuA*, 2-isopropylmalate synthase; *leuB*, 3-isopropylmalate dehydrogenase; *leuC*, 3-isopropylmalate dehydratase subunit LeuC; *leuD*, 3-isopropylmalate dehydratase subunit LeuD; *pntA*, pyridine nucleotide transhydrogenase subunit alpha; *pntB*, pyridine nucleotide transhydrogenase subunit beta; *Bcldh*, l-leucine dehydrogenase; *brnQ*, branched chain amino acid transporter BrnQ; *livJ*, Leu/Ile/Val-binding protein; *livK,* leucine-specific-binding protein; *sucAB*, alpha-ketoglutarate dehydrogenase; *pflB*, formate acetyltransferase; *ldhA*, lactate dehydrogenase A; *poxB*, pyruvate oxidase

**Fig. 2:**
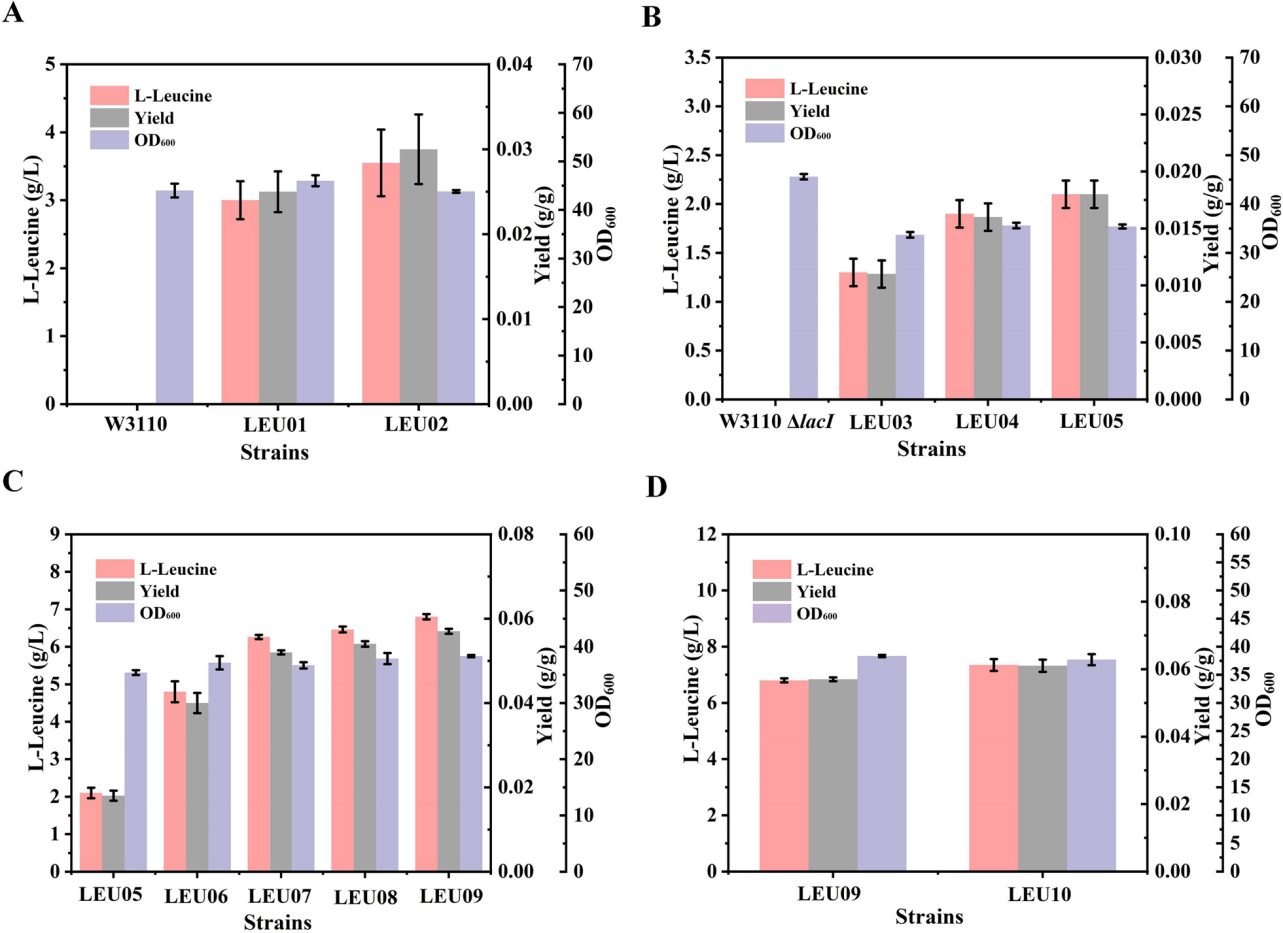
Optimization of the pathway for l-leucine biosynthesis and cell growth. **A** Introduction of 2-isopropylmalate synthase (IPMS) encoding genes from *Corynebacterium glutamicum* and *Escherichia coli*; **B** overexpression of the *leuA*^fbr^*-ecj* gene in the chromosome; **C** overexpression of l-leucine operon genes; **D** removal of the transcriptional attenuation of *leuA*

In *E. coli*, l-leucine biosynthesis starts from the precursors 2-ketoisovalerate and acetyl-CoA, and proceeds through a series of reactions catalyzed by four enzymes encoded by genes *leuA*, *leuB*, *leuC*, and *leuD*, respectively (Fig. 1). Among them, 2-isopropylmalate synthase (IPMS), the key enzyme encoding by *leuA*, has been proven to be subject to feedback inhibition by l-leucine [21]. Different feedback-resistant (fbr) IPMS mutants have been reported, including those of *C. glutamicum* and *E. coli* [11, 22]. To eliminate the inhibitory effect of the substrate on IPMS, the *leuA*^fbr^*-cgb* gene derived from *C. glutamicum* and *leuA*^fbr^*-ecj* gene derived from *E. coli* were transferred into *E. coli* W3110 using the trc promoter (P_trc_)-driven expression system, resulting in strains LEU01 and LEU02, respectively. These strains were tested by shake flask fermentation, and their growth and accumulation of l-leucine are shown in Fig. 2A. Compared to the wild-type strain with almost no l-leucine, the amount produced by the LEU01 and LEU02 strains were higher at 3 and 3.55 g/L, respectively. These results suggest that the introduction of the *leuA*^fbr^*-cgb* and *leuA*^fbr^*-ecj* genes into the host had play a positive role in releasing the feedback regulation effect of l-leucine, and this strategy provides a feasible solution to alleviate the bottleneck of its metabolic flux.

Compared with gene overexpression through plasmid cloning, chromosome integration has the advantages of the need for antibiotics being avoided and gene expression being more stable [23]. Because strain LEU02 had accumulated slightly more l-leucine than strain LEU01, the feedback-resistant *leuA*^fbr^*-ecj* gene driven by P_trc_ was introduced into *E. coli* W3110 Δ*lacI*, resulting in strains LEU03. As expected, the l-leucine titer in strain LEU03 increased to 1.3 g/L (Fig. 2B). Optimization of the enzyme expression level can effectively balance metabolism by optimizing the copy number of genes, thus improving the titer of target chemicals [24]. To enhance the expression of the rate-limiting enzyme, we attempted to integrate multiple copies of *leuA*^fbr^*-ecj* into the *E. coli* genome. Therefore, another one or two copies of *leuA*^fbr^*-ecj* were introduced into LEU03 successively, resulting in strains LEU04 and LEU05. The accumulation of l-leucine showed an increasing trend with increasing number of gene copies, with strain LEU05 carrying three copies of *leuA*, producing slightly more of the amino acid (up to 2.1 g/L) than strain LEU04. The shake flask results suggested that *leuA*^fbr^*-ecj* overexpression could increase the metabolic flux toward l-leucine synthesis, which lays a foundation for further improvement of the amino acid titer.

The non-rate-limiting genes in the l-leucine biosynthesis pathway of *E.coli* are organized in the *leuBCD* operon [25]. To strengthen the metabolic flux to l-leucine, we integrated the natural *leuBCD* operon into the *yjgX* locus of LEU05 to produce strain LEU06. The amount of l-leucine accumulated by strain LEU06 increased from 2.1 to 4.8 g/L (Fig. 2C). These results showed that strengthening the natural metabolic flux was effective in improving the synthesis of l-leucine.

2-Ketoisovalerate, an immediate precursor of l-leucine biosynthesis, is generated by acetolactate synthase through the pyruvate flux [26, 27]. The acetohydroxy acid synthase is feedback inhibited by 2-ketoisovalerate [28]; therefore, releasing this substrate feedback inhibition effect might be a favorable method to achieve better l-leucine production efficiency. The *ilvIH*^fbr^ operon (genes encoding feedback-resistant acetolactate synthase) was introduced into the *yjiT* locus of LEU06, generating strain LEU07. In addition, increase the level of 2-ketoisovalerate, the *ilvIH* and *ilvEDC* genes (encoding branched-chain-amino-acid aminotransferases) were integrated into the *ylbE* and *yjiV* locus in strain LEU07, resulting in strains LEU08 and LEU09, respectively. Consequently, the l-leucine titer reached up to 6.8 g/L, 41.67% higher than that produced by LEU06 (Fig. 2C), indicating that enriching the 2-ketoisovalerate pool promotes l-leucine biosynthesis. Over-expression of key enzyme genes in the biosynthesis pathway from pyruvate to l-leucine and optimized gene expression effectively achieved the accumulation of the target product.

### Removal of the transcriptional attenuation of *leuABCD* expression

In addition to the over-expression of crucial enzyme genes in the l-leucine biosynthesis pathway, removal of the control of transcriptional attenuation is also an effective strategy for regulating the distribution of the carbon flux. The *leuABCD* operon is regulated by leucine-mediated transcriptional attenuation in *C. glutamicum*, and the accumulation of the amino acid could be increased by replacing the promoter and attenuator sequences [29]. To evaluate the feasibility of this strategy in *E. coli*, the promoter and attenuation regions of the *leuABCD* gene in strain LEU09 were replaced with P_trc_, resulting in strain LEU10. These results showed that removal of the attenuation of *leuABCD* had raised the l-leucine titer to 7.35 g/L. This implies that the replacement of the attenuator sequence and the further enhancement of l-leucine biosynthesis genes were effective in improving synthesis of the amino acid (Fig. 2D).

### Modification of the l-leucine transport system

**Fig. 3:**
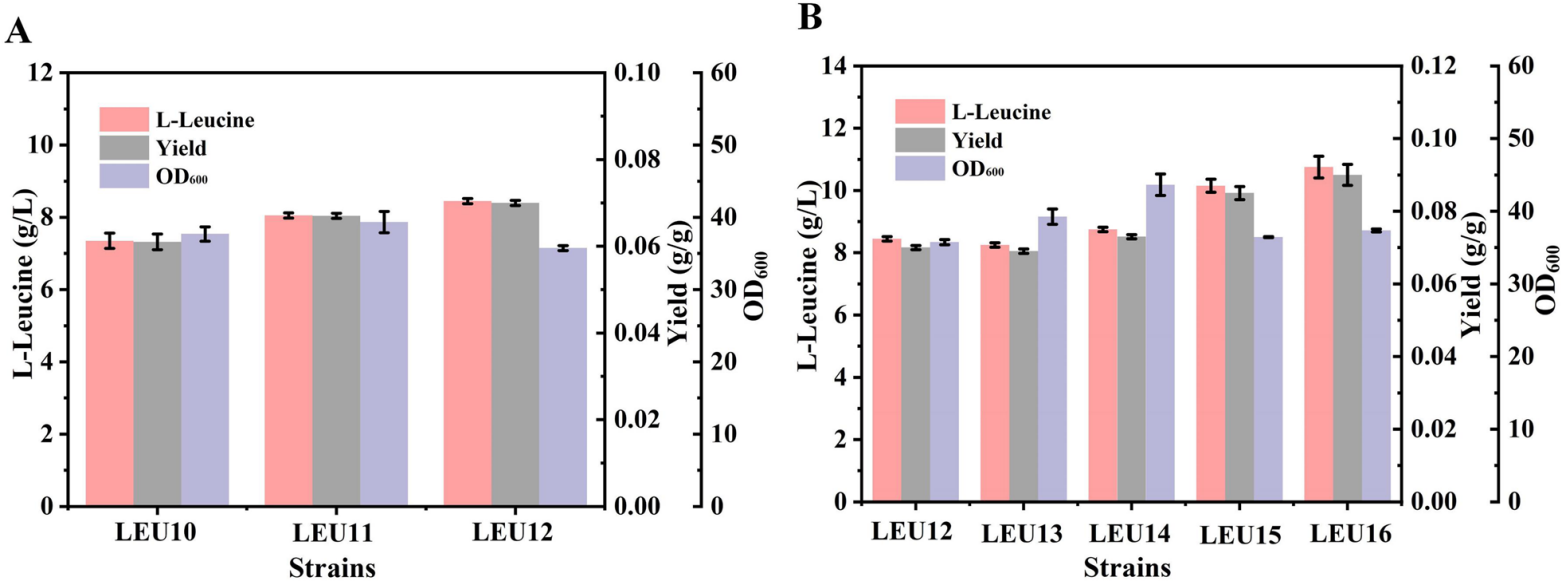
Modification of the l-leucine transport system. **A** Elimination of l-leucine reabsorption; **B** overexpression of the l-leucine export system gene

Eliminating the reabsorption of the product and promoting its outflow can result in its continuous synthesis in the cell, which is essential for the efficient production of chemicals [30]. In addition, it is worth noting that efficient l-leucine efflux will further alleviate the feedback inhibition of intracellular products. Previous researches studies have demonstrated that the l-leucine importers are controlled by leucine-specific-binding protein (LivK) and Leu/Ile/Val-binding protein (LivJ), and the gene coding for leucine efflux protein (*yeaS*) participates in the output system in *E. coli* [31–33]. Therefore, we sequentially deleted the *livK* and *livJ* genes in strain LEU10 to generate strains LEU11 and LEU12. The l-leucine production in strain LEU12 was slightly higher than that in the other strains, with 8.45 g/L produced, indicating that the elimination of reabsorption of the target product had a positive effect on its accumulation (Fig. 3A).

In addition, the branched-chain amino acid exporters BrnF and BrnE are the carriers of the l-leucine export system in *C. glutamicum* [34]. To evaluate the effect of *yeaS* and its homologous *azlC* (encoding branched-chain amino acid permease from *C. glutamicum*) and *brnFE* gene in *E. coli*, these P_trc_ driven genes were, respectively, integrated into the *livJ* locus of strain LEU11, resulting in strains LEU13, LEU14 and LEU15. Compared with strain LEU12, there was no further improvement in l-leucine production reaped through the integration of *yeaS*, whereas integration of the *brnFE* genes led to a 20.12% increase in the titer, which reached 10.15 g/L. Meanwhile, overexpression of the heterologous *azlC* gene resulted in a slightly improvement in the l-leucine titer (Fig. 3B). BrnQ, which encodes branched chain amino acid transporter, is responsible for the intake of extracellular branched chain amino acids. Therefore, we further inserted the *brnFE* genes into the *brnQ* locus of strain LEU14 to construct the LEU16 strain for subsequent genetic modification to increase the exporters. The above results reveal that an efficient transport system plays a key role in further improving the titer of l-leucine. By eliminating the absorption of l-leucine and introducing efficient exporters, the loss of carbon source was effectively avoided and the inhibition of intracellular substrate was further reduced.

### Cooperative utilization of cofactors for efficient l-leucine biosynthesis

**Fig. 4:**
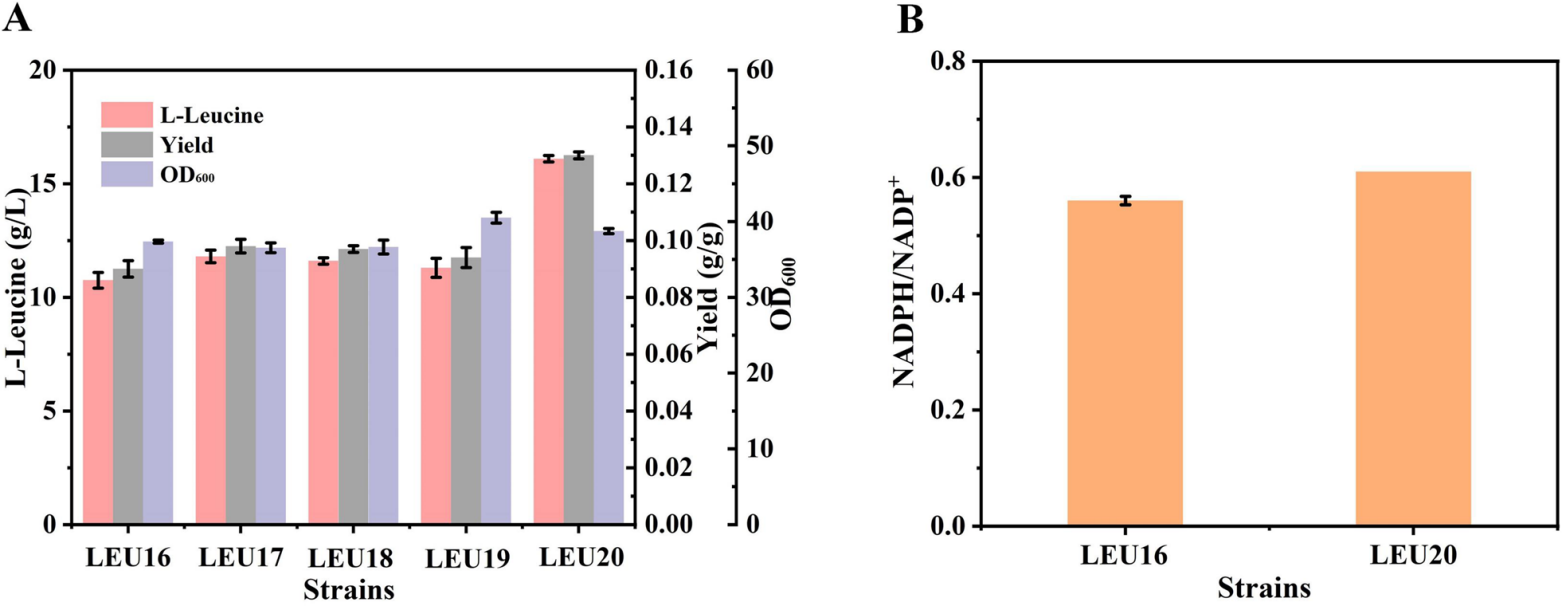
Effects of the cooperative utilization of cofactors. **A** Biomass and l-leucine titer of strains LEU16-20. **B** NADPH/NADP^+^ levels of strains LEU16 and LEU20

Intracellular redox balance is a crucial factor in the overproduction of l-leucine, as two molecules of NADPH are consumed in the biosynthesis of one molecule of the amino acid in *E. coli*, and the glycolysis pathway produces excess NADH to maintain cell metabolism [35]. Overexpression of the *pntAB* gene (coding for NAD(P) transhydrogenase), which increases intracellular NADPH levels, was successfully applied to the efficient synthesis of NADPH-dependent products [36, 37]. Besides improving the availability of NADPH, changing the cofactor demand from that of NADPH to NADH would also be an effective strategy to ensure there is a balance of intracellular cofactors [38]. We speculate that this reaction is a crucial rate-limiting step for the synthesis of l-leucine. Therefore, we hypothesized that a cooperative cofactor strategy of combining NADPH and NADH by redesigning the redox metabolic network for l-leucine production could improve the product titer. Therefore, the *pntAB* genes were introduced into the *yjiP* locus of strain LEU16, to construct strain LEU17. The l-leucine titer of LEU17 had increased to 11.8 g/L. Wang et al. replaced the endogenous glutamate dehydrogenase in *C. glutamicum* with the NADH-dependent glutamate dehydrogenase from *Bacillus subtilis* to improve the balance of cofactors in the biosynthetic pathway, thereby increasing the yield of l-leucine [13].The *rocG* gene from *B. subtilis* was inserted in the *gltB* locus of strain LEU17, generating strain LEU18*.* Unfortunately, the substitution of glutamate dehydrogenase in *E. coli* did not increase the l-leucine titer. The results showed that modifying the coenzyme requirement of glutamate dehydrogenase had no significant effect on l-leucine synthesis. The reason may be that the carbon flux of l-glutamic acid synthesis in the metabolic network is relatively weak, leading to a slight consumption of NADH, which is not conducive to the balance of intracellular cofactors. Therefore, it has no obvious promoting effect on l-leucine biosynthesis. Previously, it has been reported that engineering of leucine dehydrogenase has been established to improve chemical production [39]. To test the rationale of balancing cofactors to improve l-leucine production, we introduced heterologous l-leucine dehydrogenase for using NADH. *Esldh* gene from *Exiguobacterium sibiricum* and the *Bcldh* gene from *Bacillus cereus* were inserted in the *ilvE* locus of strain LEU17, generating strain LEU19 and LEU20, respectively. The shake flask fermentation results showed that the l-leucine titer of strain LEU19 did not increase. Surprisingly, the l-leucine titer of LEU20 had increased significantly to 16.1 g/L, which was 36.44% higher than that of the control strain (Fig. 4A). Furthermore, the NADPH/NADP^+^ level of strain LEU20 was slightly higher than that of strain LEU16 (Fig. 4B). These results indicate that overexpression of the *pntAB* genes and the substitution of l-leucine dehydrogenase from *B. cereus* had significantly improved the l-leucine titer and achieved the balance of intracellular redox.

### Suppression of the TCA cycle with a toggle switch

Although the new strain constructed to this point had achieved a certain level of l-leucine accumulation following systems metabolic engineering, there are still carbon sources utilized for normal cell growth and product synthesis that would result in a decrease in the yield and an increase in the industrial production cost. Traditionally, an engineering strategy for increasing acetyl-CoA is to attenuate citrate synthase activity using a weaker promoter. However, statically regulated protein expression levels inevitably impose a metabolic burden on cell growth [11]. To address the low l-leucine yield, we attempted to regulate the TCA cycle through the toggle switch control of *sucAB* gene expression, based on the dynamic regulatory circuit of the Esa QS system of *Pantoea stewartii*, thereby pulling the carbon flux toward the biosynthetic pathway of the target product. The transcriptional regulator EsaR^I70V^ was bound by the P_esaS_ promoter and activated transcription. With the accumulation of the signal molecule 3-oxyhexanoyl homoserine lactone (AHL), the P_*esaS*_ promoter was inactivated; that is, *sucAB* transcription was arrested. The rate of AHL accumulation in the regulatory circuit is determined by the intensity of *esaI* gene expression. The different intensities of promoter activity could trigger the time required to switch the regulatory system and further result in the appropriate intensity of *sucAB* gene expression. To prevent the overflux of carbon from pyruvate that can occur in the process of weakening the TCA cycle, we deleted the *poxB*, *pflB*, and *ldhA* genes in strain LEU20, thereby generating strain LEU21, LEU22 and LEU23 in turn.

**Fig. 5:**
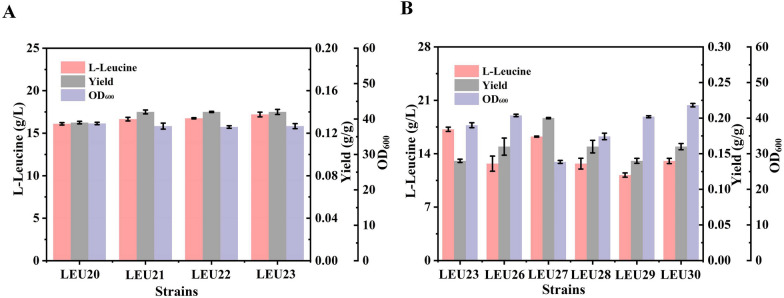
Effects of the dynamic regulation of the TCA cycle. **A** Knockout of by-products; **B** effects of the dynamic regulation of *sucAB* operon expression on l-leucine production and biomass

Subsequently, the *esaR* gene (controlled by the strong promoter P_*apFAB104*_) was introduced into the *yeep* locus of strain LEU23, and the promoter of *sucAB* was separately replaced with P_*esaS*_, generating strains LEU24 and LEU25, respectively. Next, *esaI* genes driven by promoters and RBS of different intensities (P_b__S1_, P_b__S2_, P_b__S3_, P_b__S4_ and P_b__S5_) were introduced into the strain LEU25 to facilitate AHL production, generating strains LEU26-30 [40]. As a result, the l-leucine production level of strain LEU27 had increased slightly to 16.25 g/L, and surprisingly, its conversion rate was significantly increased by 42.86% to 0.2 g/g glucose (Fig. 5). The LEU27 strain showed a decrease in cell density at 24 h, which eliminated the unexpected carbon flux transfer from pyruvate to the TCA cycle. The increase in the yield of strain LEU27 indicates that cell growth and l-leucine production can be balanced by turning off the expression of the *sucAB* genes at a more appropriate switching time.

It is well-known that there is a coupling between l-leucine production and growth, so balancing the carbon flux between them has become the important and difficult point in the construction of l-leucine production strain. In the later stage of cell growth, the switch of *sucAB* node was turned off, which effectively inhibited the metabolic flux of TCA cycle and promoted the synthesis of excess carbon to l-leucine.

### Fed-batch production of L-leucine in a bioreactor

**Fig. 6:**
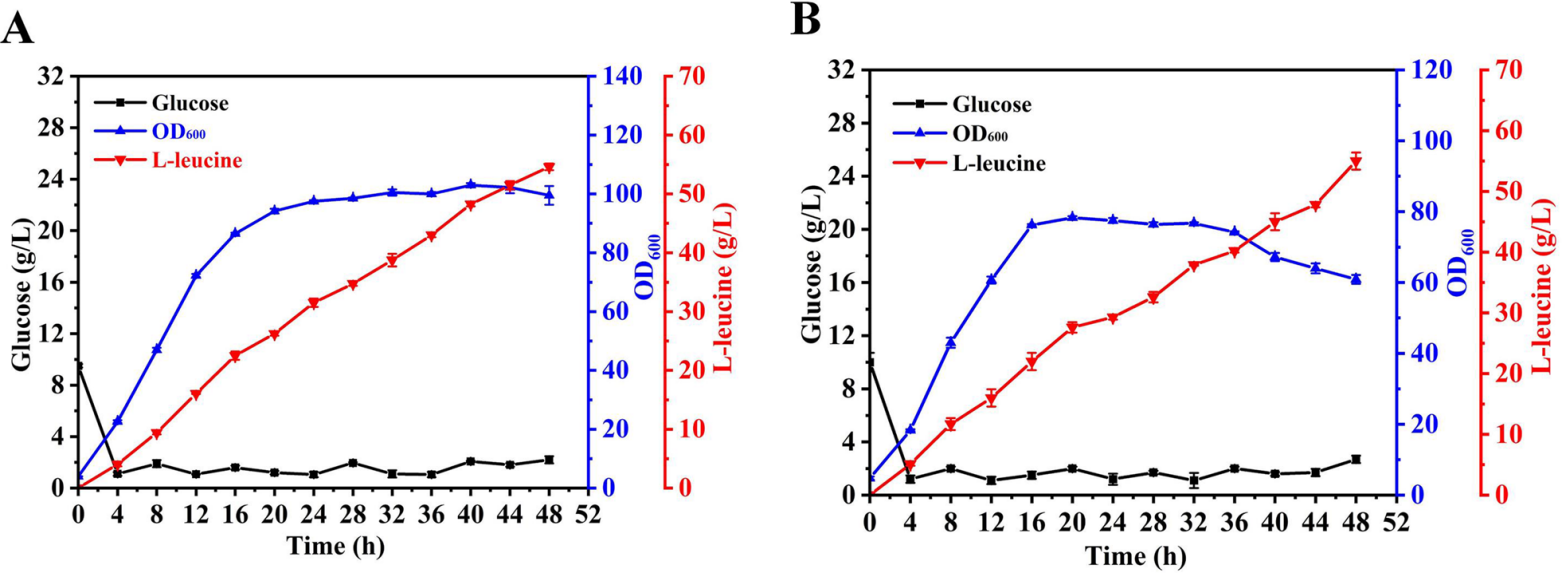
Fed-batch fermentation engineered l-leucine-overproducing strains LEU23 and LEU27. **A** Fermentation profiles of strain LEU23; **B** fermentation profiles of strain LEU27

To further evaluate the potential fermentation performance of the engineered strains, LEU23 and LEU27 were separately added to 5 L bioreactors for fed-batch fermentation. As shown in Fig. 6, strain LEU27 produced 55 g/L of l-leucine, with a yield of 0.23 g/g glucose. By comparison, the l-leucine titer and yield of strain LEU23 were 54.6 g/L and 0.17 g/g glucose, respectively. These fed batch fermentation data demonstrated that strain LEU27 accumulated slightly more l-leucine than strain LEU23. Surprisingly, the fermentation supernatant of strain LEU27 hardly had other detectable branched-chain amino acids, which would effectively reduce the cost of separation and extraction in the downstream processes of large-scale industrial production.

## Conclusion

This research article outlines the methods we had used to construct a plasmid-free nonauxotrophic l-leucine-overproducing *E. coli* strain. The metabolic engineering strategies included the (1) strengthening of the l-leucine biosynthesis; (2) enrichment of precursors pools; (3) optimization of the l-leucine transport system; and (4) cooperative utilization of cofactors. A dynamic switch for regulating the *sucAB* node was designed to weaken the metabolic carbon flux of the TCA cycle and further increase the l-leucine yield. To the best of our knowledge, strain LEU27 has produced the titer of l-leucine (55 g/L) to date. Our engineering strategy of strain modification provides a methodology for the construction of microbial cell factories capable of producing high titers of l-leucine or related products.

## Materials and methods

### Bacterial strains and plasmids

The strains constructed in this study are listed in Table 1. *E. coli* W3110 was used as the starting strain, whereas *E. coli* JM109 was used as the cloning host for plasmid construction. The pREDCas9 and pGRB plasmids and the CRISPR/Cas9 gene editing system were used for constructing the various *E. coli* strains.

**Table 1:** *Escherichia coli* strains used and constructed in this study

Strains	Characteristics	Source
JM109	Cloning host	This laboratory
W3110	Wild type, starting strain	This laboratory
LEU01	W3110, pTrc99a-*leuA*^fbr^*-cgb*	This study
LEU02	W3110, pTrc99a-*leuA*^fbr^*-ecj*	This study
LEU03	W3110, Δ*lacI*, *ycjV*::P_trc_-*leuA*^fbr^*-ecj*	This study
LEU04	LEU03, *yciQ*::P_trc_-*leuA*^fbr^*-ecj*	This study
LEU05	LEU04, *yghE*::P_trc_-*leuA*^fbr^*-ecj*	This study
LEU06	LEU05, *yjgX*::P_trc_-*leuBCD*	This study
LEU07	LEU06, *yjiT*::P_trc_*-ilvIH*^fbr^	This study
LEU08	LEU07, *ylbE*::P_trc_*-ilvIH*^fbr^	This study
LEU09	LEU08, *yjiV*::P_trc_-*ilvEDC*	This study
LEU10	LEU09, P_*leuA*_::P_trc_	This study
LEU11	LEU10, Δ*livK*	This study
LEU12	LEU11, Δ*livJ*	This study
LEU13	LEU11, *livJ*::P_trc_-*yeaS*	This study
LEU14	LEU11, *livJ*::P_trc_*-azlC*	This study
LEU15	LEU11, *livJ*::P_trc_*-brnFE*	This study
LEU16	LEU14, *brnQ*::P_trc_*-brnFE*	This study
LEU17	LEU16, *yjiP*::*pntAB*	This study
LEU18	LEU17, *gltB*::P_trc_-*rocG*	This study
LEU19	LEU17, *ilvE*::P_trc_-*Esldh*	This study
LEU20	LEU17, *ilvE*::P_trc_-*Bcldh*	This study
LEU21	LEU20, Δ*poxB*	This study
LEU22	LEU21, Δ*pflB*	This study
LEU23	LEU22, Δ*ldhA*	This study
LEU24	LEU23, *yeeP*::P_*apFAB104*_-*esaR*	This study
LEU25	LEU24, P_*sucAB*_::P_*esaS*_	This study
LEU26	LEU25, *trpR*::P_b__s1_-*esaI*	This study
LEU27	LEU25, *trpR*::P_b__s2_-*esaI*	This study
LEU28	LEU25, *trpR*::P_b__s3_-*esaI*	This study
LEU29	LEU25, *trpR*::P_b__s4_-*esaI*	This study
LEU30	LEU25, *trpR*::P_b__s5_-*esaI*	This study

### Genetic manipulations and culture conditions

Gene deletion and integration in *E. coli* were performed using the standardized protocols of the CRISPR/Cas9 gene editing method [41, 42]. The primers for gene manipulation are listed in Additional file 1: Table S1. Herein, we describe the deletion of the lactate dehydrogenase A (*ldhA*) gene as an example. First, the primers (gRNA-*ldhA*-S and gRNA-*ldhA*-A) were annealed to form dsDNA, which included a 20 bp complementary sequence and a flanking sequence homologous to the pGRB trunk. Then, the pGRB-*ldhA* plasmid was constructed through homologous recombination of the dsDNA and the linearized vector. The total DNA-*ldhA* fragment was obtained, of which the was amplified with the upstream homologous arm (primers UP-*ldhA*-S and UP-*ldhA*-A) and the downstream homologous arm (DN-*ldhA*-S and DN-*ldhA*-A). The DNA-*ldhA* and pGRB sequences were transfected into pRED–Cas9 containing cells via electrotransformation and then transformed cells were cultured on Lysogeny Broth (LB) agar plates (supplemented with spectinomycin and ampicillin) at 30 ℃. The bacterial suspension was cultured on LB medium for 16–18 h at 30 ℃, and then the positive single colony was verified by colony PCR. To cure the plasmid expressing the targeted gRNA, the positive recombinant was cultured in LB containing 0.2% l-arabinose for 14 h. Then, the bacterial solution was further cultured for 10 h in a 42 ℃ shaking incubator to lose the pRED–Cas9 plasmid. Finally, the donor DNA fragment with the target gene was incorporated into the host genome using the chromosomal integration technique. These same procedures were used for the construction of all the other strains.

### Fermentation in shake flasks

The engineered strains were first cultivated at an inclined plane, and then transferred into 30 mL of seed medium in a shake flask and cultured at 37 °C with shaking at 200 rpm. The seed medium was composed of KH_2_PO_4_ 1.2 g, yeast extract 10 g, peptone 5 g, MgSO_4_⋅7H_2_O 0.5 g, MnSO_4_ 10 mg, FeSO_4_⋅7H_2_O 10 mg, V_H_ 0.3 mg, V_B1_ 1.3 mg and glucose 20 g, per liter. Then, a 15% inoculum of the seed culture was transferred to a 500 mL baffled flask containing fermentation medium composed of KH_2_PO_4_ 2 g, yeast extract 2 g, peptone 4 g, sodium citrate dihydrate 1 g, MgSO_4_ 0.7 g, MnSO_4_ 0.1 g, V_H_ 0.2 mg, FeSO_4_ 0.1 g, V_B1_ 0.8 mg and glucose 20 g, per liter. Using phenol red as the pH indicator, NH_4_OH (25%, v/v) was added the culture medium when the latter’s pH value was less than 7. When the culture was in a state of sugar depletion, a glucose solution (60%, w/v) was provided intermittently under aseptic conditions.

### Fermentation in a 5 L bioreactor

The activated strains were first cultured in a bioreactor containing 2 L of seed medium. When the absorbance at 600 nm (OD_600_) of the seed culture broth had reached 11–15, a 15% inoculum was transferred to a 5 L bioreactor, and the temperature was set to 37 ℃. The fermentation medium of the bioreactor contained K_2_HPO_4_ 7 g, yeast extract 2 g, citric acid 2 g, (NH_4_)_2_SO_4_ 3 g, MnSO_4_⋅7H_2_O 10 mg, FeSO_4_⋅7H_2_O 30 mg, L-methionine 1 g, MgSO_4_⋅7H_2_O 1 g, V_Bx_ 0.5 mg, V_H_ 1 mg and glucose 10 g, per liter. During the fermentation process, the dissolved oxygen content was set to 20% by controlling the aeration rate and agitation speed. The pH was controlled at 6.5 through the automatic feeding of ammonium hydroxide (25%, v/v). When the sugar in the medium was exhausted, glucose (80%, w/v) was added automatically, so that its final concentration was not higher than 3 g/L.

### Analytical methods

During the fermentation process, the cell density was measured using an ultraviolet spectrophotometer to detect the OD_600_. The glucose concentration was measured using an SBA-40C biosensor (Shandong Province Academy of Sciences, China). The l-leucine content was determined using high-performance liquid chromatography, with an acetonitrile/water mixture (50:50, v/v) and 50 mM sodium acetate used to prepare the mobile phase. The data presented in this study represent the average and standard deviation of three independent samples. The data presented in this study represent the mean and standard deviation of three independent cultures.

### Supplementary Information

**Additional file 1: Table S1.** Primers employed in this study.

## Data Availability

All data generated or analyzed during this study are included in this published article and its additional files.
