# Supplementary material for: Construction of a plasmid-free l-leucine overproducing Escherichia coli strain through reprogramming of the metabolic flux
Source: Biotechnol Biofuels Bioprod. 2023 Sep 29;16:145. doi: 10.1186/s13068-023-02397-x (PMC10541719; doi:10.1186/s13068-023-02397-x)
Supplement: Supplementary file 1 — Additional file 1: Table S1. Primers employed in this study. [file 13068_2023_2397_MOESM1_ESM.docx]

Table S1 Primers employed in this study.

| Primer | Sequence (5′-3′) |
| --- | --- |
| pTrc99a*-cgb-*S | TGGCTGGCGGCGTTTAAGTCGACCTGCAGGCATGCAAG |
| pTrc99a*-cgb-*A | GATGAATGCATCGTTAGGAGACATGAATTCCATGGTCTGTTTCCTGT |
| *leuA^fbr^-cgb-*S | ACAGGAAACAGACCATGGAATTCATGTCTCCTAACGATGCATTCATC |
| *leuA^fbr^-cgb-*A | CTTGCATGCCTGCAGGTCGACTTAAACGCCGCCAGCCA |
| pTrc99a*-ecj-*S | AAACAACAAGGAAACCGTGTGAGTCGACCTGCAGGCATGCAAG |
| pTrc99a*-ecj-*A | GAAAATAATGACTTGCTGGCTCATGAATTCCATGGTCTGTTTCCTGT |
| *leuA^fbr^-ecj-*S | ACAGGAAACAGACCATGGAATTCATGAGCCAGCAAGTCATTATTTTC |
| *leuA^fbr^-ecj-*A | CTTGCATGCCTGCAGGTCGACTCACACGGTTTCCTTGTTGTTT |
| UP*-ycjV-*S | GAATGCGCCGACGATTTTAG |
| UP*-ycjV-*A | AATTGTTATCCGCTCACAATTCCACACATTATACGAGCCGGATGATTAATTGTCAAGCCGACAAACACGATGAACTC |
| *leuA-*S1 | TCCGGCTCGTATAATGTGTGGAATTGTGAGCGGATAACAATTTCACACAGGAAACAGACCATGAGCCAGCAAGTCATTATTTTC |
| *leuA-*A1 | GGGTCACGTAGATCATGGTGGTCACACGGTTTCCTTGTTGTTTTCGTTGT |
| DN*-ycjV-*S | ACAACGAAAACAACAAGGAAACCgtgTGACCACCATGATCTACGTGACCC |
| DN*-ycjV-*A | CACCAGCACGGACCACTAACT |
| UP*-yciQ-*S | TTACTTGAAGCATTGGGCGAAC |
| UP*-yciQ-*A | AATTGTTATCCGCTCACAATTCCACACATTATACGAGCCGGATGATTAATTGTCAACCAGTCAAGATGCCAGGGTTC |
| *leuA-*S2 | TCCGGCTCGTATAATGTGTGGAATTGTGAGCGGATAACAATTTCACACAGGAAACAGACCATGAGCCAGCAAGTCATTATTTTC |
| *leuA-*A2 | AGGATTTGCTGGTTCTTGTCAGACTCACACGGTTTCCTTGTTGTTTTCGTTGT |
| DN*-yciQ-*S | ACAACGAAAACAACAAGGAAACCGTGTGAGTCTGACAAGAACCAGCAAATCCT |
| DN*-yciQ-*A | ATAGCTTCACCGTGGGCATAAC |
| UP*-yghE-*S | GTCAGGCACTGGCGAAAGAT |
| UP*-yghE-*A | AATTGTTATCCGCTCACAATTCCACACATTATACGAGCCGGATGATTAATTGTCAACGCAAGCCATAAACCCACA |
| *leuA-*S3 | TCCGGCTCGTATAATGTGTGGAATTGTGAGCGGATAACAATTTCACACAGGAAACAGACCATGAGCCAGCAAGTCATTATTTTC |
| *leuA-*A3 | ACGCATTTCGATGTCGGAATCACACGGTTTCCTTGTTGTTTTCGTTGTG |
| DN*-yghE-*S | CACAACGAAAACAACAAGGAAACCGTGTGATTCCGACATCGAAATGCGT |
| DN*-yghE-*A | AGGCGTTGTTGTGGCAGATT |
| UP*-yjgX-*S | GGAAGTCAACGGGTTATGCG |
| UP*-yjgX-*A | AATTGTTATCCGCTCACAATTCCACACATTATACGAGCCGGATGATTAATTGTCAAAAAATCACCACGAATACCAGAATC |
| *leuBCD-*S1 | TCCGGCTCGTATAATGTGTGGAATTGTGAGCGGATAACAATTTCACACAGGAAACAGACCATGTCGAAGAATTACCATATTGCC |
| *leuBCD-*A1 | GCCCCAAGGGGTTATGCTAGCCTACAAATTGAGTTATGTTCATTTAAATATGATGTTGTTCAGTTACACCCCTTCTGCTACATAGC |
| *leuBCD-*S2 | CTGAACAACATCATATTTAAATGAACATAACTCAATTTGTAGGCTAGCATAACCCCTTGGGGCACAGTGTCTTCCCTGAGCCG |
| *leuBCD-*S3 | GCAAGCGTCGCCACAAAG |
| *leuBCD-*A3 | CGGCTCAGGGAAGACACTGTTTAATTCATAAACGCAGGTTGTTTT |
| DN*-yjgX-*S | AAAACAACCTGCGTTTATGAATTAAACAGTGTCTTCCCTGAGCCG |
| DN*-yjgX-*A | GGCGAAGGATACCATCAAGC |
| UP*-leuA-*S | CAGGTTGCGACATTCCCAG |
| UP*-leuA-*A | AATTGTTATCCGCTCACAATTCCACACATTATACGAGCCGGATGATTAATTGTCAAGATGATTGAGTATTCGCGGTA |
| DN*-leuA-*S | TCCGGCTCGTATAATGTGTGGAATTGTGAGCGGATAACAATTTCACACAGGAAACAGACCATGAGCCAGCAAGTCATTATTTTCG |
| DN*-leuA-*A | CATGGTGTAGCCCACGGTGTC |
| UP*-yjiT-*S | AATAGTTGTTGCCGCCTGAGT |
| UP*-yjiT-*A | AATTGTTATCCGCTCACAATTCCACACATTATACGAGCCGGATGATTAATTGTCAAAAAACAGGCAGCAAAGTCCC |
| *ilvIH^fbr^-*S1 | TCCGGCTCGTATAATGTGTGGAATTGTGAGCGGATAACAATTTCACACAGGAAACAGACCATGGAGATGTTGTCTGGAGCC |
| *ilvIH^fbr^-*A1 | ACATCCCTTCACAGGTAGTGCTTTCAACGCATTATTTTATCGCCG |
| DN*-yjiT-*S | CGGCGATAAAATAATGCGTTGAAAGCACTACCTGTGAAGGGATGT |
| DN*-yjiT-*A | CAGGGCTTCCACAGTCACAAT |
| UP*-ylbE-*S | ACCCAACCTTACGCAACCAG |
| UP*-ylbE-*A | AATTGTTATCCGCTCACAATTCCACACATTATACGAGCCGGATGATTAATTGTCAATTGTTCGATAACCGCAGCAT |
| *ilvIH^fbr^-*S2 | TCCGGCTCGTATAATGTGTGGAATTGTGAGCGGATAACAATTTCACACAGGAAACAGACCATGGAGATGTTGTCTGGAGCC |
| *ilvIH^fbr^-*A2 | TTCAAAGCACGCCAGCGTCAACGCATTATTTTATCGCCG |
| DN*-ylbE-*S | CGGCGATAAAATAATGCGTTGACGCTGGCGTGCTTTGAA |
| DN*-ylbE-*A | GGCGTAACTCAGCAGGCAG |
| UP*-yjiV-*S | GTGCTGGAGGGATGATTGTTG |
| UP*-yjiV-*A | ATTGTTATCCGCTCACAATTCCACACATTATACGAGCCGGATGATTAATTGTCAACGCAGTACTTCCTGCTGGCT |
| *ilvEDC-*S1 | TCCGGCTCGTATAATGTGTGGAATTGTGAGCGGATAACAATTTCACACAGGAAACAGACCATGACCACGAAGAAAGCTGATTAC |
| *ilvEDC-*A1 | GCCTACAAATTGAGTTATGTTCATTTAAATATGATGTTGTTCAGTTAACCCCCCAGTTTCGATTT |
| *ilvEDC-*S2 | ATGAACATAACTCAATTTGTAGGCTAGCATAACCCCTTGGGGCCGAATTACCGGCGTTCACTATA |
| *ilvEDC-*A2 | TCACCTCCACCAGCACATCC |
| *ilvEDC-*S3 | AACGGCTGCATCGTGAAAACGGCAGG |
| *ilvEDC-*A3 | GATTCAGTGTATTGAAGTAGTTAGCCATTTAACCCCCCAGTTTCGATTT |
| *ilvEDC-*S4 | AAATCGAAACTGGGGGGTTAAATGGCTAACTACTTCAATACACTGAATC |
| *ilvEDC-*A4 | TATAGTGAACGCCGGTAATTCGTTAACCCGCAACAGCAATACG |
| DN*-yjiV-*S | CGTATTGCTGTTGCGGGTTAACGAATTACCGGCGTTCACTATA |
| DN*-yjiV-*A | TCACCTCCACCAGCACATCC |
| UP*-livK-*S | AAGTGGAACCAGTGTAGCGAAAT |
| UP*-livK-*A | ATCCTGGTCATAGCGTTTTGGATCCCCATTCGTGATGTTGTG |
| DN*-livK-*S | CACAACATCACGAATGGGGATCCAAAACGCTATGACCAGGAT |
| DN*-livK-*A | GTAGGTACTGCCCAGCGTGAC |
| UP*-livJ-*S | ATTCTCAGGATGAACATAAAGGGTA |
| UP*-livJ-*A1 | GTAGGTGGTCCAAACGAATGCCGTAGATGTCAGACGCAGGC |
| DN*-livJ-*S | GCCTGCGTCTGACATCTACGGCATTCGTTTGGACCACCTAC |
| DN*-livJ-*A | GTAACAATCCTGGCGTTTCAT |
| UP*-livJ-*A2 | AATTGTTATCCGCTCACAATTCCACACATTATACGAGCCGGATGATTAATTGTCAACGTAGATGTCAGACGCAGGC |
| *yeaS*-S | TCCGGCTCGTATAATGTGTGGAATTGTGAGCGGATAACAATTTCACACAGGAAACAGACCGTGTTCGCTGAATACGGGGT |
| *yeaS*-A | GTAGGTGGTCCAAACGAATGCTCAGGATTGCAGCGTCGC |
| DN*-livJ-*S2 | GCGACGCTGCAATCCTGAGCATTCGTTTGGACCACCTAC |
| UP*-livJ-*A3 | AATTGTTATCCGCTCACAATTCCACACATTATACGAGCCGGATGATTAATTGTCAAAAAACGTAGATGTCAGACGCAGGC |
| *azlC*-S | TCCGGCTCGTATAATGTGTGGAATTGTGAGCGGATAACAATTTCACACAGGAAACAGACCATGAGTTCCGAAAATTTAAAAGAAA |
| *azlC*-A | GTAGGTGGTCCAAACGAATGCTCACTCATGGGAAGTCCTGATCT |
| DN*-livJ-*S3 | AGATCAGGACTTCCCATGAGTGAGCATTCGTTTGGACCACCTAC |
| UP*-livJ-*A4 | AATTGTTATCCGCTCACAATTCCACACATTATACGAGCCGGATGATTAATTGTCAAAAAACGTAGATGTCAGACGCAGGC |
| *brnFE*-S1 | TCCGGCTCGTATAATGTGTGGAATTGTGAGCGGATAACAATTTCACACAGGAAACAGACCGTGCAAAAAACGCAAGAGATTC |
| *brnFE*-A1 | GTAGGTGGTCCAAACGAATGCTTAGAAAAGATTCACCAGTCCAACA |
| DN*-livJ-*S4 | TGTTGGACTGGTGAATCTTTTCTAAGCATTCGTTTGGACCACCTAC |
| UP*-brnQ-*S | CGTTTCAGTTTTGTTATCCCG |
| UP*-brnQ-*A | AATTGTTATCCGCTCACAATTCCACACATTATACGAGCCGGATGATTAATTGTCAATAAAGCCCAGAGCGATGATAT |
| *brnFE*-S2 | TCCGGCTCGTATAATGTGTGGAATTGTGAGCGGATAACAATTTCACACAGGAAACAGACCGTGCAAAAAACGCAAGAGATTC |
| *brnFE*-A2 | GCAACAGACAGGATGACCAGCTTAGAAAAGATTCACCAGTCCAACA |
| DN*-brnQ-*S | TGTTGGACTGGTGAATCTTTTCTAAGCTGGTCATCCTGTCTGTTGC |
| DN*-brnQ-*A | CCAGGCAGGCGATGAAGA |
| UP*-yjiP-*S | GCCATACCGCCAGCAAGAT |
| UP*-yjiP-*A | GCAAGGCGAAGGATTATTTTTGCAGATATTCCCCTTTCCACC |
| *pntAB*-S1 | GGTGGAAAGGGGAATATCTGCAAAAATAATCCTTCGCCTTGC |
| *pntAB*-A1 | GCCCCAAGGGGTTATGCTAGCCTACAAATTGAGTTATGTTCATTTAAATATGATGTTGTTCAGTTAATTTTTGCGGAACATTTTCA |
| *pntAB*-S2 | CTGAACAACATCATATTTAAATGAACATAACTCAATTTGTAGGCTAGCATAACCCCTTGGGGCGACGGATGACAAACGCAAAGC |
| *pntAB*-A2 | AAAGGCGGATTTTTACTGTGGA |
| *pntAB*-S3 | CGTTGCGCCGAAAGAATT |
| *pntAB*-A3 | GCTTTGCGTTTGTCATCCGTCTTACAGAGCTTTCAGGATTGCAT |
| DN-*yjiP*-S | ATGCAATCCTGAAAGCTCTGTAAGACGGATGACAAACGCAAAGC |
| DN-*yjiP*-A | AAAGGCGGATTTTTACTGTGGA |
| UP*-gltB-*S | GCTCACCGCCTGCTGATAC |
| UP*-gltB-*A | AATTGTTATCCGCTCACAATTCCACACATTATACGAGCCGGATGATTAATTGTCAAGCATTTTATACTGCCTTAATTGGTA |
| *rocG*-S | TCCGGCTCGTATAATGTGTGGAATTGTGAGCGGATAACAATTTCACACAGGAAACAGACCATGTCAGCAAAGCAAGTCTCGA |
| *rocG*-A | GCATCCCGACAGCGTAGTTTTTAGACCCATCCGCGGAA |
| DN*-gltB-*S | TTCCGCGGATGGGTCTAAAAACTACGCTGTCGGGATGC |
| DN*-gltB-*A | AGGTCAGGGATAAGCGGTGT |
| UP*-ilvE-*S | GATGAACATTCCTGGCGTTAC |
| UP*-ilvE-*A1 | AATTGTTATCCGCTCACAATTCCACACATTATACGAGCCGGATGATTAATTGTCAACGCTTCAACCTGGTCTTTCC |
| *Esldh*-S | TCCGGCTCGTATAATGTGTGGAATTGTGAGCGGATAACAATTTCACACAGGAAACAGACCATGGTGGAAACCAATGTGGAAG |
| *Esldh*-A | CAGGAGGAAACCATCGCATCTTAGCCGCGACTGCCCAG |
| DN*-ilvE-*S1 | CTGGGCAGTCGCGGCTAAGATGCGATGGTTTCCTCCTG |
| DN*-ilvE-*A | CGCCAACCTGAATACCGTCTA |
| UP*-ilvE-*A2 | AATTGTTATCCGCTCACAATTCCACACATTATACGAGCCGGATGATTAATTGTCAACGCTTCAACCTGGTCTTTCC |
| *Bcldh*-S | TCCGGCTCGTATAATGTGTGGAATTGTGAGCGGATAACAATTTCACACAGGAAACAGACCATGACATTAGAAATCTTCGAATACTTAG |
| *Bcldh*-A | CAGGAGGAAACCATCGCATCTTAGCGACGGCTAATAATATCGT |
| DN*-ilvE-*S2 | ACGATATTATTAGCCGTCGCTAAGATGCGATGGTTTCCTCCTG |
| UP*-poxB-*S | GTGTTTGCGGCTGCTGTAAG |
| UP*-poxB-*A | AGGAAATCGCCCATCAACAGCCAGTTCTCCGCTAAGTTGT |
| DN*-poxB-*S | ACAACTTAGCGGAGAACTGGCTGTTGATGGGCGATTTCCT |
| DN*-poxB-*A | ACCTTAGCCAGTTTGTTTTCG |
| UP-*pflB*-S | CGGTTTTGAGCACAGTATCGC |
| UP-*pflB*-A | GCAAGCAATAGCGTAGTCATCGAAGGACTCGTCACCCTCGTA |
| DN-*pflB*-S | TACGAGGGTGACGAGTCCTTCGATGACTACGCTATTGCTTGC |
| DN-*pflB*-A | CGGGTATTCGCCTTCG |
| UP*-ldhA-*S | CGAGCGGTAGCCAGATGC |
| UP*-ldhA-*A | GTTCGTTCGGGCAGGTTTCTACCGCTTCGCAGCCATT |
| DN*-ldhA-*S | AATGGCTGCGAAGCGGTAGAAACCTGCCCGAACGAAC |
| DN*-ldhA-*A | ATGCTGCCGGAAATCATCA |
| UP*-yeeP-*S | GGTCAGGAGGTAACTTATCAGCG |
| UP*-yeeP-*A | GGATCCTTTCTCCTCTTTAATGAATTGATGGCTGTAAGTATCCTATAGGTTAGACTTTATGTCGAATGGCAGGGCTCCGTTTT |
| *esaR*-S | CAGCCATCAATTCATTAAAGAGGAGAAAGGATCCATGTTTTCTTTTTTCCTTGAAAATC |
| *esaR*-A | ACAGGTTCAGAAGAAAATCCAGTTCTCACTACCTGGCCGCTGAC |
| DN*-yeeP-*S | GTCAGCGGCCAGGTAGTGAGAACTGGATTTTCTTCTGAACCTGT |
| DN*-yeeP-*A | ACGATGTCAGCAGCCAGCA |
| UP*-sucA-*S | TGTGATTCGCCCGCTGCCAGGTT |
| UP*-sucA-*A | GGATACGCTTACACTGTTGTGAGCACGGTTTACGCATTACGTTGC |
| P_eas_*-*S | GCAACGTAATGCGTAAACCGTGCTCACAACAGTGTAAGCGTATCC |
| P_eas_*-*A | TTCAAAGCGCTGTTCTGCATGGATCCTTTCTCCTCTTTAATGAATTG |
| DN*-sucA-*S | CAATTCATTAAAGAGGAGAAAGGATCCATGCAGAACAGCGCTTTGAA |
| DN*-sucA-*A | GCAGTAGGTTTGCTTGAGGG |
| UP*-trpR-*S | GCGACCCATACGGTGAAGAT |
| UP*-trpR-*A1 | GCTAGCACAGTCCCTAGGACTGAGCTAGCTGTAAAGCTGAATAGGGTGATTGTTGG |
| *esaI*-S1 | TTTACAGCTAGCTCAGTCCTAGGGACTGTGCTAGCAAGGAGATATACCATGCTTGAACTGTTTGACGTCAGT |
| *esaI*-A | GTAGGACGGATAAGGCGTTCATTATCAGACCGGCAGCGTC |
| DN*-trpR-*S | GACGCTGCCGGTCTGATAATGAACGCCTTATCCGTCCTAC |
| DN*-trpR-*A | AATGCCCGCCGTTTACTTC |
| UP*-trpR-*A2 | GCTAGCATAGTACCTAGGACTGAGCTAGCCGTAAAGCTGAATAGGGTGATTGTTGG |
| *esaI*-S2 | TTTACGGCTAGCTCAGTCCTAGGTACTATGCTAGCAAGGAGATATACCATGCTTGAACTGTTTGACGTCAGT |
| UP*-trpR-*A3 | GCTAGCACTATACCTAGGACTGAGCTAGCCGTAAAGCTGAATAGGGTGATTGTTGG |
| *esaI*-S3 | TTTACGGCTAGCTCAGTCCTAGGTATAGTGCTAGCAGGAAACAGACCATGCTTGAACTGTTTGACGTCAGT |
| UP*-trpR-*A4 | GCTAGCATAATACCTAGGACTGAGCTAGCTGTAAAGCTGAATAGGGTGATTGTTGG |
| *esaI*-S4 | TTTACAGCTAGCTCAGTCCTAGGTATTATGCTAGCAGGAAACAGACCATGCTTGAACTGTTTGACGTCAGT |
| UP*-trpR-*A5 | GCTAGCACTGTACCTAGGACTGAGCTAGCCGTCAAGCTGAATAGGGTGATTGTTGG |
| *esaI*-S5 | TTGACGGCTAGCTCAGTCCTAGGTACAGTGCTAGCAGGAAACAGACCATGCTTGAACTGTTTGACGTCAGT |
| gRNA*-ycjV-*S | AGTCCTAGGTATAATACTAGTCGAGTTTTTCACCTTCTGCGGTTTTAGAGCTAGAA |
| gRNA*-ycjV-*A | TTCTAGCTCTAAAACCGCAGAAGGTGAAAAACTCGACTAGTATTATACCTAGGACT |
| gRNA-*yciQ-*S | AGTCCTAGGTATAATACTAGTCAGGAAAAACGCAAAACCATGTTTTAGAGCTAGAA |
| gRNA*-yciQ-*A | TTCTAGCTCTAAAACATGGTTTTGCGTTTTTCCTGACTAGTATTATACCTAGGACT |
| gRNA*-yghE-*S | AGTCCTAGGTATAATACTAGTTCTGCCGCATCGATGAATGCGTTTTAGAGCTAGAA |
| gRNA*-yghE-*A | TTCTAGCTCTAAAACGCATTCATCGATGCGGCAGAACTAGTATTATACCTAGGACT |
| gRNA*-yjgX-*S | AGTCCTAGGTATAATACTAGTTCGCGACCACCGTAACTGGCGTTTTAGAGCTAGAA |
| gRNA*-yjgX-*A | TTCTAGCTCTAAAACGCCAGTTACGGTGGTCGCGAACTAGTATTATACCTAGGACT |
| gRNA*-leuA-*S | AGTCCTAGGTATAATACTAGTGGGACGTTTTTATTGCGTCAGTTTTAGAGCTAGAA |
| gRNA*-leuA-*A | TTCTAGCTCTAAAACTGACGCAATAAAAACGTCCCACTAGTATTATACCTAGGACT |
| gRNA*-yjiT-*S | AGTCCTAGGTATAATACTAGTAGGGATTATGAACGGCAATGGTTTTAGAGCTAGAA |
| gRNA*-yjiT-*A | TTCTAGCTCTAAAACCATTGCCGTTCATAATCCCTACTAGTATTATACCTAGGACT |
| gRNA*-ylbE-*S | AGTCCTAGGTATAATACTAGTACACTGGCTGGATGTGCAACGTTTTAGAGCTAGAA |
| gRNA*-ylbE-*A | TTCTAGCTCTAAAACGTTGCACATCCAGCCAGTGTACTAGTATTATACCTAGGACT |
| gRNA*-yjiV-*S | AGTCCTAGGTATAATACTAGTATCCCGCATTTCTTAAAGTCGTTTTAGAGCTAGAA |
| gRNA*-yjiV-*A | TTCTAGCTCTAAAACGACTTTAAGAAATGCGGGATACTAGTATTATACCTAGGACT |
| gRNA*-livK-*S | AGTCCTAGGTATAATACTAGTGTCCGGCCCGATTGCCCAGTGTTTTAGAGCTAGAA |
| gRNA*-livK-*A | TTCTAGCTCTAAAACACTGGGCAATCGGGCCGGACACTAGTATTATACCTAGGACT |
| gRNA*-livJ-*S | AGTCCTAGGTATAATACTAGTCACCCCAGCGGCAACCGCGCGTTTTAGAGCTAGAA |
| gRNA*-livJ-*A | TTCTAGCTCTAAAACGCGCGGTTGCCGCTGGGGTGACTAGTATTATACCTAGGACT |
| gRNA*-brnQ-*S | AGTCCTAGGTATAATACTAGTTACAGGCAGGCGAACACGTCGTTTTAGAGCTAGAA |
| gRNA*-brnQ-*A | TTCTAGCTCTAAAACGACGTGTTCGCCTGCCTGTAACTAGTATTATACCTAGGACT |
| gRNA*-yjiP-*S | AGTCCTAGGTATAATACTAGTGTAAAGACCCGCGAAGGCGAGTTTTAGAGCTAGAA |
| gRNA*-yjiP-*A | TTCTAGCTCTAAAACTCGCCTTCGCGGGTCTTTACACTAGTATTATACCTAGGACT |
| gRNA*-gltB-*S | AGTCCTAGGTATAATACTAGTAACACACCTTTATGACAGTCGTTTTAGAGCTAGAA |
| gRNA*-gltB-*A | TTCTAGCTCTAAAACGACTGTCATAAAGGTGTGTTACTAGTATTATACCTAGGACT |
| gRNA-*ilvE-*S | AGTCCTAGGTATAATACTAGTTCGACGAACTTGAGAACGTCGTTTTAGAGCTAGAA |
| gRNA*-ilvE-*A | TTCTAGCTCTAAAACGACGTTCTCAAGTTCGTCGAACTAGTATTATACCTAGGACT |
| gRNA*-poxB-*S | AGTCCTAGGTATAATACTAGTTTGCCACCGCAATCACGTTCGTTTTAGAGCTAGAA |
| gRNA*-poxB-*A | TTCTAGCTCTAAAACGAACGTGATTGCGGTGGCAAACTAGTATTATACCTAGGACT |
| gRNA*-pflB-*S | AGTCCTAGGTATAATACTAGTCTGAAGCGACCACCACCCTGGTTTTAGAGCTAGAA |
| gRNA*-pflB-*A | TTCTAGCTCTAAAACCAGGGTGGTGGTCGCTTCAGACTAGTATTATACCTAGGACT |
| gRNA-*ldhA*-S | AGTCCTAGGTATAATACTAGTCTGGAAGAGCTGAAAAAGCAGTTTTAGAGCTAGAA |
| gRNA-*ldhA*-A | TTCTAGCTCTAAAACTGCTTTTTCAGCTCTTCCAGACTAGTATTATACCTAGGACT |
| gRNA*-yeeP-*S | AGTCCTAGGTATAATACTAGTACAGAATATTCGCGAAAAAAGTTTTAGAGCTAGAA |
| gRNA*-yeeP-*A | TTCTAGCTCTAAAACTTTTTTCGCGAATATTCTGTACTAGTATTATACCTAGGACT |
| gRNA*-trpR-*S | AGTCCTAGGTATAATACTAGTCAGATGAGCGCGAAGCGTTGGTTTTAGAGCTAGAA |
| gRNA*-trpR-*A | TTCTAGCTCTAAAACCAACGCTTCGCGCTCATCTGACTAGTATTATACCTAGGACT |
